# Supplementary material for: The Protective Effects of IL-31RA Deficiency During Bleomycin-Induced Pulmonary Fibrosis
Source: Front Immunol. 2021 Mar 19;12:645717. doi: 10.3389/fimmu.2021.645717 (PMC8017338; doi:10.3389/fimmu.2021.645717)
Supplement: Supplementary file 1 [file Data_Sheet_1.PDF]

Supplementary Material

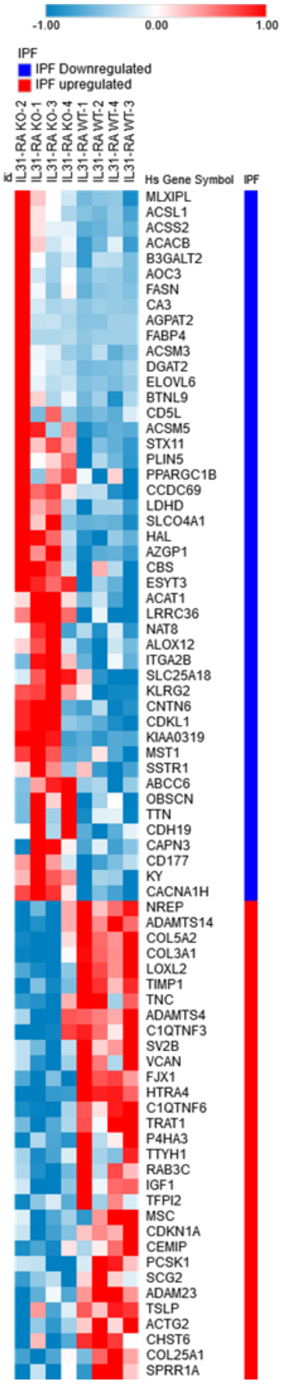

Supplementary Figure 1. Heatmap representation of total 77 genes that correlated between IPF and the knockdown of IL-31RA during bleomycin-induced pulmonary fibrosis.

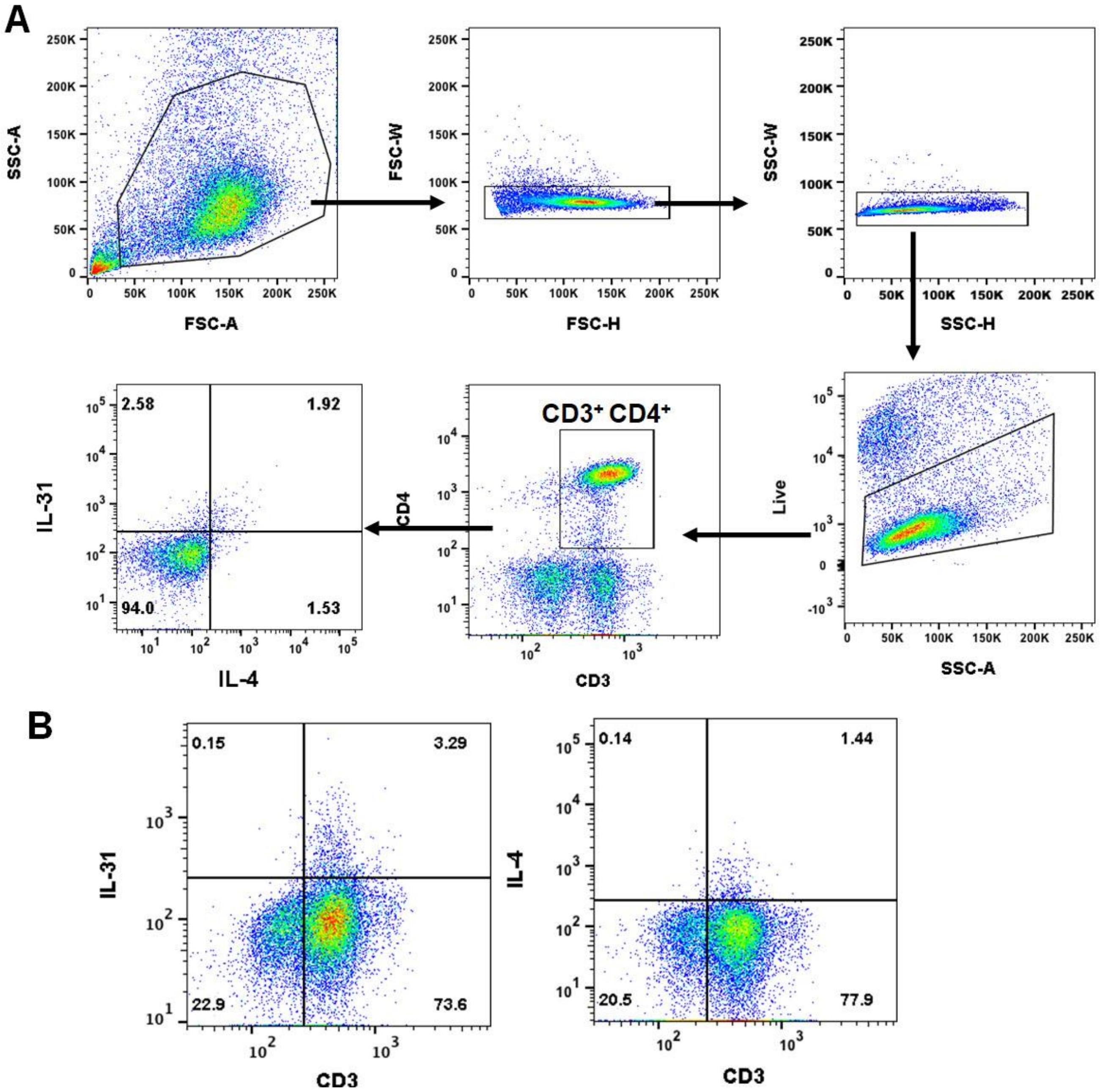

Supplementary Figure 2. Flow cytometry analysis of IL-31-positive T cells in PBMCs. (A) Gating strategy used to characterize IL-31-positive cells in PBMC from IPF and healthy subjects (Related to Figures 5C & 5D). (B) The representative images showing CD3 T cells as a major cell type that produce IL-31 or IL-4 in PBMCs of IPF patients.

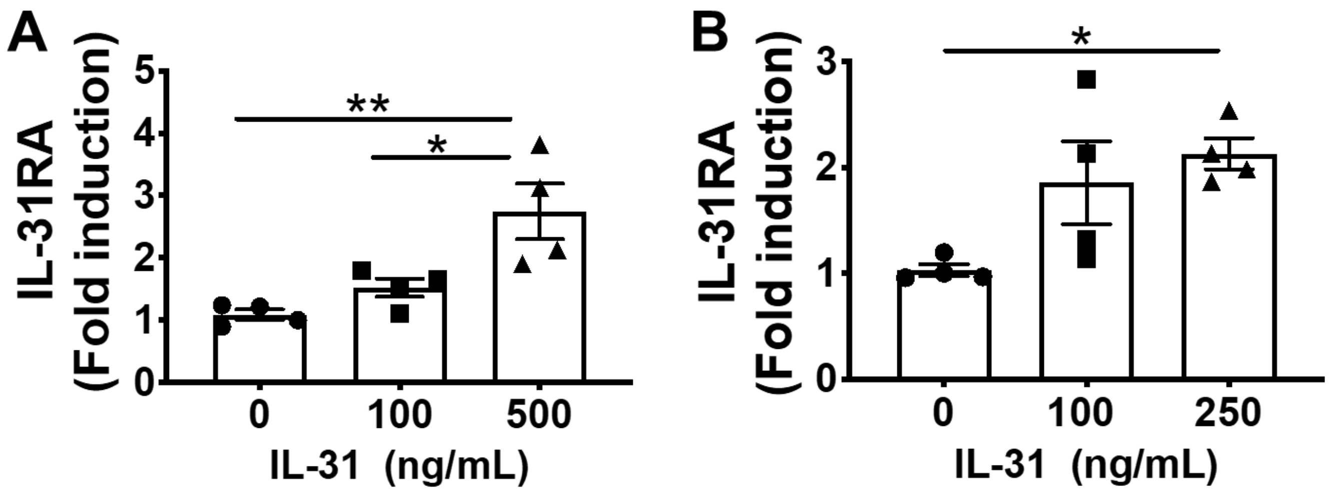

Supplementary Figure 3. IL-31 induces the expression of IL-31RA in human airway epithelial cells. BEAS 2B cells (A) and primary human bronchial epithelial (NHBE) cells (B) were stimulated with recombinant IL-31 for 24 hours and the transcripts of IL-31RA were measured using RT-PCR. Data presented as Mean $\pm$ SEM (n=4/group). Statistical analysis was performed using one-way ANOVA with Tukey's multiple comparisons test * $p<0.05$; ** $p<0.005$.

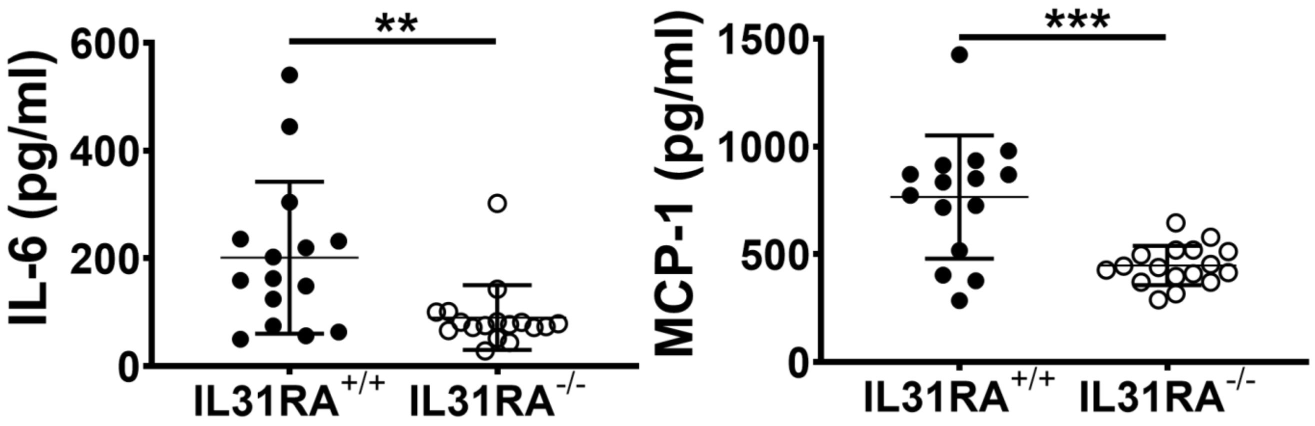

Supplementary Figure 4. IL-31RA deficiency resulted in reduced IL-6 and MCP-1 protein levels in the lungs during bleomycin-induced pulmonary fibrosis. Wildtype and IL31RA knock-out mice were treated intradermally with bleomycin to induce pulmonary fibrosis. Lung lysates were prepared and the protein levels of IL-6 and MCP-1 were determined by ELISA (R&D Systems). Cumulative data of two independent experiments are presented as mean $\pm$ SD. N= 15-17/group. An unpaired t-test was used to compare the difference between groups. ** $p<0.005$; *** $p<0.0005$

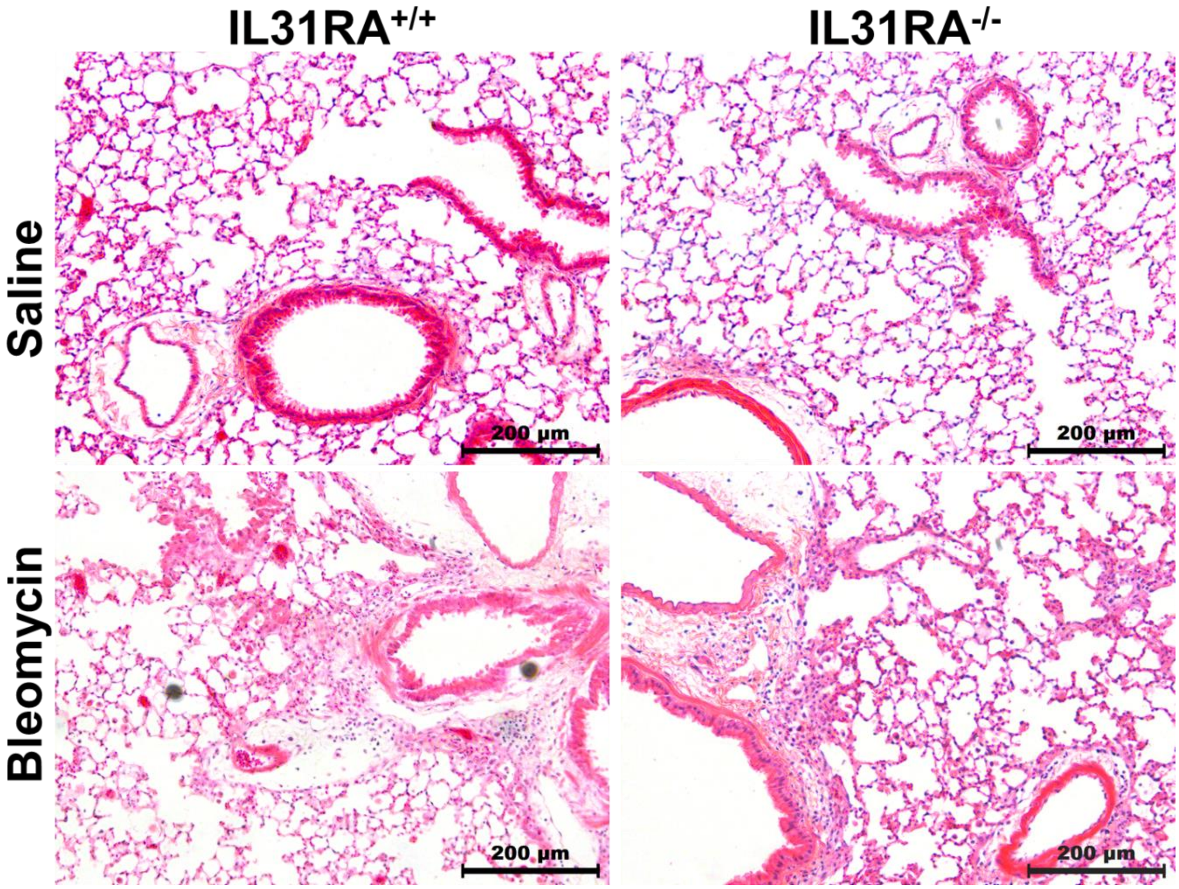

Supplementary Figure 5. The loss of IL-31RA signaling resulted in limited or no changes in tissue inflammation during bleomycin-induced pulmonary fibrosis. wildtype (IL-31RA$^{+/+}$) and IL-31RA knockout (IL31RA$^{-/-}$) mice were treated intradermally with bleomycin or saline for four weeks to induce pulmonary fibrosis. Lung sections of saline or bleomycin-treated mice were stained with H & E. Scale bar 200 µm.

Supplementary Table 1. List of primers used for RT-PCR.

Gene ID	Forward primer	Reverse primer
**mIL6**	TCCAGTTGCCTTCTTGGGAC	GTGTAATTAAGCCTCCGACTTG
**HRPT**	GCCCTTGACTATAATGAGTACTTCAGG	TTCAACTTGCGCTCATCTTAGG
**mCol1a1**	AGACATGTTCAGCTTTGTGGAC	GCAGCTGACTTCAGGGATG
**mCol3a1**	TCCCCTGGAATCTGTGAATC	TGAGTCGAATTGGGGAGAAT
**mFn1**	CGGAGAGAGTGCCCCTACTA	CGATATTGGTGAATCGCAGA
**mMMP-13**	CTGGCACACGCTTTTCCTCCTG	GGGCTGGGTCACACTTCTCTGGT
**mTimp1**	GCAAAGAGCTTTCTCAAAGACC	AGGGATAGATAAACAGGGAAACACT
**mKrt5**	CAGAGCTGAGGAACATGCAG	CATTCTCAGCCGTGGTACG
**mKrt14**	ATCGAGGACCTGAAGAGCAA	TCGATCTGCAGGAGGACATT
**hMCP1**	GCTCATAGCAGCCACCTTCA	ACAATGGTCTTGAAGATCACAGC
**hIL-6**	CAGGAGCCCAGCTATGAACT	GAAGGCAGCAGGCAACAC

Supplementary Table 2. Clinical parametres of de-identified human samples (IPF and healthy controls).

**Clinical Parameters**	**IPF**	**Control**
**Number (N)**	**7**	**8**
**Sex**	**3F, 3M**	**4F, 4M**
**Age (mean)**	**61**	**54**

Supplementary Table 3. The list of genes differentially expressed in IL-31RA knockout mice during bleomycin-induced pulmonary fibrosis.

Genes Upregulated in IL-31RA KO Mice			Genes Downregulated in IL-31RA KO Mice		
**Gene Symbol**	**Log2 Fold**	**p-Val**		**Log2 Fold**	**p-Val**
**Chil4**	**2.673613**	**0.000142**	**Sox18**	**-0.58571**	**6.6E-08**
**4933400F21Rik**	**2.449708**	**0.002679**	**Psapl1**	**-0.59137**	**0.013305**
**A330074K22Rik**	**2.196978**	**0.000553**	**Gjb5**	**-0.59174**	**0.005489**
**AI593442**	**1.968621**	**0.000628**	**Col4a2**	**-0.59288**	**8.66E-10**
**Clca1**	**1.909314**	**4.68E-10**	**Carmn**	**-0.59383**	**0.023858**
**5830411N06Rik**	**1.822686**	**0.00576**	**Nrcam**	**-0.59543**	**3.96E-06**
**Krt79**	**1.752798**	**1.81E-06**	**Tmem45a**	**-0.59679**	**6.92E-08**
**Fabp12**	**1.731421**	**0.001595**	**Adamts2**	**-0.59682**	**1.01E-07**
**Olfr750**	**1.717942**	**0.026154**	**Ccl2**	**-0.59743**	**0.000452**
**Adgrf3**	**1.591881**	**0.010797**	**Id3**	**-0.59908**	**3.42E-12**
**Leap2**	**1.569674**	**0.027845**	**Pcdhb19**	**-0.59999**	**0.016732**
**Sult2a1**	**1.553427**	**0.049406**	**Fhl2**	**-0.60137**	**0.000547**
**Tmem178b**	**1.529699**	**0.004423**	**Csmd1**	**-0.60385**	**0.017902**
**Hsd17b13**	**1.525838**	**0.029312**	**Klhdc8a**	**-0.60541**	**4.06E-06**
**Ngp**	**1.522654**	**0.02251**	**Chodl**	**-0.60661**	**0.004555**
**Lctl**	**1.519688**	**0.036391**	**Lhx6**	**-0.60747**	**2.73E-05**
**Mir7013**	**1.517201**	**0.011605**	**Bmper**	**-0.6082**	**3.86E-05**

**Fcgbp**	**1.509645**	**1.37E-08**	**Prss35**	**-0.61025**	**0.004394**
**Cd300e**	**1.506774**	**9.07E-08**	**Alox15**	**-0.6106**	**0.035185**
**Cyp1a1**	**1.477192**	**5.82E-05**	**Apcdd1**	**-0.61292**	**2.27E-05**
**Pkdrej**	**1.472519**	**0.00013**	**Galnt16**	**-0.61366**	**0.000523**
**Slc6a20a**	**1.471947**	**1.48E-11**	**Ccdc80**	**-0.6146**	**1.98E-08**
**Ceacam10**	**1.455468**	**0.015381**	**Nrg1**	**-0.61622**	**1.43E-05**
**Ces2c**	**1.437188**	**0.049946**	**Fstl1**	**-0.61667**	**3.06E-10**
**Hist1h4h**	**1.409537**	**1.33E-07**	**Tnfrsf12a**	**-0.6173**	**5.58E-07**
**4930433N12Rik**	**1.394948**	**0.006936**	**P2ry14**	**-0.61762**	**2.28E-08**
**Colq**	**1.390389**	**4.14E-05**	**Wscd2**	**-0.61825**	**0.004752**
**C730036E19Rik**	**1.37155**	**0.001619**	**Disp2**	**-0.61923**	**4.36E-09**
**Entpd8**	**1.288417**	**0.00099**	**Baalc**	**-0.61984**	**0.027398**
**Ccl20**	**1.28541**	**0.010986**	**Adgrb2**	**-0.62059**	**0.020691**
**Rpph1**	**1.284619**	**0.016616**	**Alpl**	**-0.62141**	**0.002383**
**Art2b**	**1.265003**	**0.022463**	**Col5a2**	**-0.62162**	**3.05E-08**
**Gm15713**	**1.25535**	**0.034382**	**Nxpe5**	**-0.62429**	**0.003603**
**Gm16863**	**1.254123**	**0.041035**	**Junb**	**-0.6263**	**1.63E-06**
**Tbata**	**1.239877**	**0.000767**	**Tnc**	**-0.62762**	**1.92E-05**
**Cst8**	**1.225231**	**0.023483**	**Sfrp1**	**-0.6279**	**8.35E-09**
**Muc5ac**	**1.215486**	**7.82E-11**	**Aplnr**	**-0.62936**	**0.000942**

**9230117E06Rik**	**1.212656**	**0.029542**	**Adamts4**	**-0.63026**	**7.53E-09**
**Lrat**	**1.212301**	**2.85E-09**	**Mt1**	**-0.63152**	**0.00417**
**Gria1**	**1.209916**	**1.2E-05**	**Rgs4**	**-0.6325**	**2.11E-06**
**Slc4a1**	**1.20287**	**0.004447**	**Acta2**	**-0.63381**	**6.64E-05**
**Tecrl**	**1.201598**	**1.15E-06**	**Serpinb9b**	**-0.63412**	**3.59E-05**
**Gypa**	**1.192801**	**0.010383**	**Sema7a**	**-0.63563**	**5.2E-07**
**Slc7a10**	**1.186105**	**1.28E-08**	**Ramp3**	**-0.63749**	**0.00338**
**Stac**	**1.18021**	**0.046521**	**Gm13889**	**-0.6394**	**3.26E-07**
**Gbp10**	**1.179485**	**0.004405**	**S100a14**	**-0.6394**	**3.29E-09**
**Gpr33**	**1.164263**	**0.007738**	**Fkbp5**	**-0.6421**	**0.000412**
**Btnl9**	**1.149575**	**0.012233**	**Col1a1**	**-0.64445**	**6.06E-06**
**Kcnu1**	**1.144672**	**0.001337**	**Aspn**	**-0.64457**	**7.53E-10**
**Grip2**	**1.142652**	**0.006848**	**Mark1**	**-0.64586**	**7.1E-08**
**Gm29508**	**1.132961**	**0.027103**	**Shf**	**-0.64659**	**0.009229**
**Acoxl**	**1.125048**	**2.95E-13**	**Rtn4rl2**	**-0.64811**	**0.028092**
**Rmrp**	**1.120263**	**0.023983**	**Fam181b**	**-0.64979**	**0.005833**
**Gjb1**	**1.0942**	**0.006782**	**Cers3**	**-0.6533**	**0.000359**
**Lipf**	**1.092653**	**1.41E-05**	**Il20rb**	**-0.65552**	**0.000226**
**Cdh16**	**1.09093**	**0.006542**	**2200002D01Rik**	**-0.6596**	**9.24E-05**
**Ifi214**	**1.089664**	**0.003476**	**Actg2**	**-0.65998**	**0.00106**

**Adgre4**	**1.087422**	**3.41E-05**	**Bhlhe22**	**-0.66353**	**0.003501**
**Gm15441**	**1.085195**	**0.041732**	**Aldh1a3**	**-0.66583**	**0.003011**
**Bex2**	**1.081454**	**0.003401**	**Mboat2**	**-0.66797**	**5.34E-07**
**Cecr6**	**1.078146**	**0.024324**	**Serpina3n**	**-0.66852**	**2.33E-06**
**Gc**	**1.070781**	**0.029874**	**Fgf2**	**-0.67303**	**0.000454**
**Aass**	**1.070057**	**1.53E-08**	**Bean1**	**-0.67317**	**0.005458**
**Nrn1**	**1.061699**	**2.14E-07**	**Ptx3**	**-0.67402**	**0.01925**
**Slc1a2**	**1.047459**	**9.76E-05**	**1500015O10Rik**	**-0.67547**	**0.022997**
**Apol11b**	**1.041322**	**0.008488**	**Clec10a**	**-0.68523**	**7.38E-05**
**A930006K02Rik**	**1.039347**	**0.000676**	**Vwa1**	**-0.68636**	**1.89E-06**
**Ntn5**	**1.037847**	**0.01952**	**P4ha3**	**-0.68752**	**0.00017**
**Sbk2**	**1.030719**	**0.018273**	**Tnfrsf11b**	**-0.6895**	**2.14E-05**
**Slfn5os**	**1.029082**	**0.030258**	**Wisp1**	**-0.69371**	**6.3E-06**
**6330407A03Rik**	**1.028617**	**0.037364**	**Arg1**	**-0.6942**	**0.008545**
**Cd177**	**1.015409**	**0.020052**	**Gper1**	**-0.69561**	**0.003943**
**S1pr5**	**1.009543**	**1.04E-05**	**Cnn1**	**-0.69567**	**0.003332**
**Hc**	**1.006359**	**1.3E-21**	**Arc**	**-0.69687**	**0.0076**
**Gjb6**	**1.004205**	**2.01E-06**	**Ms4a4a**	**-0.7054**	**6.87E-05**
**1700128F08Rik**	**1.00216**	**0.00939**	**Rtn4r**	**-0.7085**	**0.046516**
**Snca**	**0.994158**	**2.63E-05**	**Ceacam16**	**-0.70922**	**0.027628**

**Ubd**	**0.983973**	**0.042134**	**Tubb3**	**-0.71689**	**0.003071**
**Gm14085**	**0.981087**	**0.01794**	**Mgp**	**-0.71866**	**4.23E-13**
**Tat**	**0.966249**	**0.036146**	**Ptgdr2**	**-0.72169**	**0.038499**
**Hmgcs2**	**0.965869**	**2.64E-08**	**Tagln**	**-0.72528**	**3.43E-08**
**Cxcr1**	**0.952686**	**0.000319**	**Stc2**	**-0.72709**	**0.001155**
**Tex11**	**0.952675**	**2.06E-08**	**Dbn1**	**-0.73075**	**8.1E-09**
**Heatr9**	**0.952508**	**0.026835**	**Saa3**	**-0.73137**	**0.019986**
**Capn9**	**0.951696**	**0.040205**	**Tubb2b**	**-0.73241**	**0.00177**
**Avpr1a**	**0.942627**	**0.009621**	**Mycn**	**-0.73252**	**0.020154**
**Cntn4**	**0.9392**	**0.003044**	**Wnk3**	**-0.7364**	**0.031459**
**Pigr**	**0.938587**	**1.45E-16**	**Flnc**	**-0.74101**	**7.69E-05**
**Itih1**	**0.925434**	**0.047826**	**Clrn1**	**-0.74147**	**0.041874**
**Ppbp**	**0.920734**	**3.31E-06**	**Syt13**	**-0.74749**	**0.024204**
**Ccr3**	**0.920423**	**0.03171**	**Rnf152**	**-0.74784**	**2.03E-05**
**Treml4**	**0.920215**	**1.7E-05**	**Cldn4**	**-0.7508**	**1.37E-08**
**Cyp26b1**	**0.918901**	**0.047801**	**Fmod**	**-0.75119**	**3E-08**
**Itih4**	**0.915934**	**8.47E-18**	**Ltbp2**	**-0.75761**	**2.11E-09**
**4930512B01Rik**	**0.909073**	**0.030949**	**Mnd1**	**-0.75825**	**0.001818**
**Vnn3**	**0.90534**	**0.000768**	**Hoxd8**	**-0.76167**	**0.01932**
**4931406H21Rik**	**0.902503**	**0.006289**	**Gal3st2**	**-0.76357**	**0.015301**

**Lrrc17**	**0.90239**	**0.000154**	**Igsf1**	**-0.7699**	**0.011721**
**Cd8a**	**0.901348**	**0.00126**	**Inhba**	**-0.77317**	**2.03E-11**
**Ctcfl**	**0.899915**	**0.005459**	**Gdf6**	**-0.77325**	**0.000578**
**Klre1**	**0.895238**	**0.000822**	**Shc4**	**-0.77344**	**0.000206**
**Awat2**	**0.885709**	**0.025555**	**Cd248**	**-0.77527**	**6.91E-13**
**Proz**	**0.867426**	**0.001534**	**Rasl10b**	**-0.78815**	**0.00022**
**Bpifa2**	**0.865212**	**0.003172**	**Fn1**	**-0.78956**	**1.17E-11**
**Chil1**	**0.863091**	**2.97E-25**	**Xirp2**	**-0.78966**	**0.005784**
**Acod1**	**0.863042**	**0.010323**	**Pcdhgb8**	**-0.79073**	**0.013895**
**Ros1**	**0.86182**	**0.000668**	**Scn8a**	**-0.79558**	**0.015527**
**Gm2115**	**0.860746**	**0.003023**	**Snord47**	**-0.80094**	**0.010658**
**Cd163l1**	**0.85727**	**0.004771**	**Dkk2**	**-0.8121**	**0.000905**
**Hpcal4**	**0.854495**	**0.000294**	**Tram1l1**	**-0.81561**	**0.005958**
**Fxyd7**	**0.853736**	**0.029262**	**Mmp13**	**-0.82059**	**7.79E-06**
**Cth**	**0.851512**	**2.17E-05**	**Tmem252**	**-0.82195**	**0.001738**
**Gm6654**	**0.848027**	**0.014921**	**Dio2**	**-0.82808**	**0.000365**
**Mgam**	**0.845967**	**0.026191**	**Smpd3**	**-0.83053**	**1.48E-06**
**Slc13a4**	**0.830348**	**0.00078**	**C130080G10Rik**	**-0.83197**	**0.012641**
**Slfn14**	**0.822254**	**0.04703**	**Col28a1**	**-0.83301**	**5.96E-10**
**Hkdc1**	**0.820164**	**0.00248**	**Lox**	**-0.83378**	**3.78E-14**

**Kcnh4**	**0.818558**	**0.032971**	**Prg4**	**-0.84001**	**6.89E-05**
**Phf24**	**0.818176**	**1.96E-05**	**Aicda**	**-0.84513**	**0.031228**
**Galnt13**	**0.817111**	**0.000362**	**Ackr1**	**-0.84516**	**0.001684**
**A930001A20Rik**	**0.815555**	**0.013087**	**Iglon5**	**-0.84796**	**0.009439**
**Mbl1**	**0.80788**	**0.021332**	**Syt8**	**-0.85556**	**0.022212**
**Tff2**	**0.805843**	**2.28E-05**	**Il22ra2**	**-0.8665**	**0.001877**
**Nalcn**	**0.802981**	**0.010329**	**Kcnh6**	**-0.86707**	**0.040281**
**Mme**	**0.802494**	**4.09E-14**	**Kcnj10**	**-0.87587**	**0.003001**
**Dio1**	**0.800167**	**0.009147**	**Fcna**	**-0.87819**	**0.000291**
**Pbld2**	**0.798502**	**4.33E-05**	**Prss22**	**-0.8832**	**0.007636**
**Cyp2ab1**	**0.797159**	**0.006177**	**Gpr176**	**-0.89271**	**2.73E-06**
**Bcan**	**0.796814**	**0.010578**	**Pappa**	**-0.8938**	**1.47E-07**
**Sh2d1a**	**0.791745**	**0.012118**	**Ccl12**	**-0.89815**	**2.82E-05**
**Dnah7c**	**0.790012**	**0.009572**	**Cd5l**	**-0.89933**	**0.033353**
**Grik4**	**0.788655**	**0.031628**	**Htra4**	**-0.90532**	**4.16E-07**
**Vipr2**	**0.786055**	**1.35E-07**	**Padi1**	**-0.90562**	**0.037523**
**Lmntd1**	**0.784217**	**0.014322**	**Snai1**	**-0.90715**	**5.46E-13**
**Gm20257**	**0.7818**	**0.001175**	**Prrx2**	**-0.90869**	**0.032842**
**D030025P21Rik**	**0.772405**	**0.029944**	**Calcr**	**-0.90955**	**0.036381**
**Pisd-ps1**	**0.771626**	**2.15E-15**	**Eln**	**-0.91772**	**3.64E-13**

**Ptgs2os2**	**0.771197**	**0.000237**	**Slc17a2**	**-0.92535**	**0.039815**
**Bend6**	**0.767975**	**0.002298**	**Xcl1**	**-0.92751**	**0.020171**
**Ppp2r2c**	**0.762647**	**0.002091**	**Wfdc12**	**-0.96427**	**0.000174**
**Arhgef38**	**0.758907**	**0.005284**	**Timp1**	**-0.96694**	**3.14E-11**
**Tubb1**	**0.751273**	**0.000656**	**Spock3**	**-0.97767**	**0.03904**
**Rgs7**	**0.750917**	**0.046371**	**Zbtb16**	**-0.98012**	**4.34E-08**
**Srpk3**	**0.750212**	**0.00254**	**Ptk6**	**-0.98222**	**0.000669**
**1810044D09Rik**	**0.747929**	**0.027686**	**Mapk4**	**-0.99586**	**0.00122**
**Cd8b1**	**0.745079**	**0.020092**	**Fbln2**	**-0.99785**	**2.95E-23**
**Gm32014**	**0.743705**	**0.028476**	**Il6**	**-0.99836**	**0.036906**
**Plppr4**	**0.74287**	**0.026068**	**Mest**	**-1.0017**	**1.93E-11**
**Agtr1b**	**0.742029**	**0.033589**	**Olfr558**	**-1.00651**	**0.025765**
**2310040G24Rik**	**0.734871**	**0.008535**	**Tceal3**	**-1.00911**	**0.045607**
**1110020A21Rik**	**0.733519**	**0.016566**	**Mmp10**	**-1.01046**	**0.009625**
**Samd15**	**0.733279**	**0.024111**	**Nts**	**-1.02542**	**0.003267**
**Hepacam**	**0.730381**	**0.022396**	**Nrxn1**	**-1.03011**	**1.44E-11**
**Krt23**	**0.729252**	**1.1E-06**	**Serpina3m**	**-1.03079**	**0.002163**
**Snora81**	**0.728818**	**0.033893**	**Pla1a**	**-1.03092**	**3.5E-14**
**Marco**	**0.726037**	**2.01E-07**	**Rnf165**	**-1.03457**	**0.006203**
**Crispld1**	**0.723924**	**0.003837**	**Cd300c**	**-1.03803**	**0.023686**

**Pisd-ps2**	**0.711691**	**1.9E-10**	**Nr1h4**	**-1.03811**	**0.028219**
**Gpr18**	**0.711572**	**0.003812**	**Resp18**	**-1.03899**	**0.003886**
**Acot1**	**0.706935**	**7.47E-06**	**Rgs16**	**-1.03921**	**2.41E-09**
**Cntfr**	**0.704008**	**0.0114**	**Hspb7**	**-1.06488**	**6.62E-06**
**S100g**	**0.703372**	**1.07E-09**	**Rgs5**	**-1.06665**	**8.08E-28**
**Nrg2**	**0.697571**	**0.004323**	**Synpo2l**	**-1.07442**	**0.001955**
**1810041L15Rik**	**0.695114**	**0.007446**	**Gjb4**	**-1.0857**	**0.010558**
**Pcdhga4**	**0.695099**	**0.00405**	**Olfr78**	**-1.09502**	**0.00636**
**Sgpp2**	**0.694221**	**2.4E-15**	**Tarm1**	**-1.10022**	**0.021312**
**Tlr11**	**0.692812**	**0.019532**	**Ereg**	**-1.10422**	**0.000217**
**Gm20597**	**0.692353**	**0.045531**	**A2m**	**-1.10641**	**0.016**
**Myo15**	**0.69213**	**0.009936**	**Egfem1**	**-1.11408**	**3.23E-07**
**Ar**	**0.691972**	**4.18E-05**	**Prtn3**	**-1.1192**	**0.026912**
**Kcna2**	**0.68989**	**0.000989**	**Tfap2a**	**-1.12856**	**4E-05**
**Cttnbp2**	**0.689289**	**0.00124**	**3300005D01Rik**	**-1.13515**	**0.007248**
**Idi1**	**0.688066**	**3.36E-11**	**Mchr1**	**-1.13824**	**0.006389**
**Spta1**	**0.685678**	**0.022097**	**Slc24a1**	**-1.14122**	**0.026726**
**B230217C12Rik**	**0.68486**	**0.011896**	**Tpsb2**	**-1.14391**	**0.04054**
**Esrrg**	**0.683934**	**0.000145**	**2210408I21Rik**	**-1.14567**	**3.64E-05**
**Lamp3**	**0.681293**	**5.48E-18**	**Fabp7**	**-1.15544**	**0.000257**

**Slc22a3**	**0.67869**	**0.03921**	**Dspp**	**-1.15652**	**0.001355**
**Aadac**	**0.675362**	**0.011606**	**Mt2**	**-1.1627**	**1.64E-06**
**Ces1f**	**0.674206**	**0.002186**	**Peg3**	**-1.17064**	**1.59E-16**
**9330159F19Rik**	**0.673698**	**0.001874**	**Dio3os**	**-1.18736**	**0.013563**
**Dpp6**	**0.670869**	**0.002994**	**Serpinb2**	**-1.19177**	**0.000283**
**Edn3**	**0.667307**	**8.1E-05**	**Krt5**	**-1.1923**	**0.002317**
**Cpm**	**0.666875**	**3.47E-14**	**Atp13a5**	**-1.19331**	**0.007924**
**Nr3c2**	**0.665198**	**2.29E-08**	**Xirp1**	**-1.20639**	**0.037702**
**Gm11744**	**0.662299**	**0.026446**	**Ankrd34a**	**-1.20732**	**0.037274**
**Tcf23**	**0.658968**	**0.010798**	**Dok5**	**-1.21049**	**0.033988**
**Esr2**	**0.655701**	**0.003412**	**Ankrd34b**	**-1.21207**	**0.000979**
**Itk**	**0.653062**	**0.001474**	**Grem1**	**-1.21501**	**0.000758**
**2610307P16Rik**	**0.652735**	**0.015737**	**Cdh3**	**-1.23744**	**2.08E-07**
**Inmt**	**0.6498**	**1.21E-06**	**Nog**	**-1.25027**	**2.96E-05**
**Alas2**	**0.649058**	**0.001107**	**Wnt10a**	**-1.25514**	**7.52E-05**
**Slc38a5**	**0.648871**	**0.00025**	**Angptl7**	**-1.26904**	**3.13E-06**
**Nkg7**	**0.647346**	**0.002451**	**Moxd1**	**-1.28491**	**1.19E-06**
**Mfsd2a**	**0.645631**	**4.61E-07**	**Fst**	**-1.306**	**2.89E-12**
**Slco4c1**	**0.64364**	**2.3E-07**	**Calcb**	**-1.3085**	**2.1E-05**
**Adra2a**	**0.643116**	**0.010946**	**Chl1**	**-1.31269**	**1.83E-15**

**Lrp2**	**0.637266**	**5.62E-11**	**Frzb**	**-1.31456**	**2.76E-06**
**5330413P13Rik**	**0.63491**	**0.007508**	**4930546K05Rik**	**-1.32176**	**0.021605**
**Snhg11**	**0.633916**	**8.75E-09**	**Zfp985**	**-1.33229**	**0.000194**
**Zpbp**	**0.630773**	**0.036032**	**Rspo4**	**-1.36**	**0.036528**
**Gm12250**	**0.630132**	**1.2E-05**	**Pgbd5**	**-1.36586**	**0.000363**
**Gm5084**	**0.630091**	**0.029315**	**Krt4**	**-1.37121**	**0.012891**
**Dcdc2a**	**0.629278**	**0.000477**	**Serpinb5**	**-1.3773**	**0.041347**
**Gpt**	**0.62757**	**5.27E-07**	**Dio3**	**-1.38034**	**1.48E-07**
**2010005H15Rik**	**0.622056**	**0.039898**	**Gcnt4**	**-1.40666**	**0.000159**
**Colgalt2**	**0.621817**	**0.000869**	**Bpifa1**	**-1.40944**	**7.56E-12**
**Sfxn4**	**0.620845**	**0.002212**	**Kcnt1**	**-1.42272**	**0.011289**
**Ptpn20**	**0.619916**	**0.006704**	**Kng2**	**-1.42341**	**1.13E-07**
**Alox12**	**0.617404**	**0.001851**	**Fgf23**	**-1.42466**	**0.018705**
**Wdr95**	**0.617079**	**0.004089**	**Kcng2**	**-1.46575**	**0.024785**
**Plin1**	**0.617052**	**0.010172**	**Pnma2**	**-1.4763**	**0.001024**
**Egfl6**	**0.61703**	**3.24E-12**	**Krt20**	**-1.52728**	**0.03796**
**2810459M11Rik**	**0.614825**	**0.034506**	**Gm12709**	**-1.55574**	**0.029477**
**Nipal1**	**0.612598**	**0.00139**	**Krt14**	**-1.57194**	**0.000799**
**Gzmb**	**0.611966**	**0.03493**	**Esm1**	**-1.60428**	**4.26E-14**
**Cd19**	**0.610868**	**0.006056**	**Ccl28**	**-1.61613**	**9.19E-06**

**Adra1a**	**0.609117**	**0.001359**	**Npas3**	**-1.61739**	**0.004178**
**H2-K2**	**0.607889**	**0.007169**	**Krt17**	**-1.65181**	**0.000267**
**Pla2g4f**	**0.604119**	**0.00071**	**C430002N11Rik**	**-1.66975**	**0.002388**
**Sftpa1**	**0.602931**	**7.46E-13**	**Tmprss11g**	**-1.6972**	**7.12E-05**
**Akap14**	**0.594143**	**0.003335**	**Tnfsf18**	**-1.74337**	**3.9E-05**
**Faim2**	**0.593782**	**0.041433**	**Cwh43**	**-1.77127**	**3.31E-05**
**Plekhd1os**	**0.593636**	**0.047726**	**Nppa**	**-1.79786**	**0.004488**
**Ric3**	**0.592527**	**0.002016**	**Calcoco2**	**-1.85255**	**0.006209**
**Cntn1**	**0.589763**	**0.049646**	**Cemip**	**-1.89049**	**1.7E-13**
**Dhtkd1**	**0.588409**	**0.017916**	**Krt6a**	**-1.94824**	**0.013342**
**C530008M17Rik**	**0.586904**	**0.012049**	**Pkp1**	**-2.02817**	**9.69E-06**
			**Tmem267**	**-2.78047**	**1.03E-48**
			**Erdr1**	**-4.59138**	**6.42E-20**
